# Localization of contrast-enhanced breast lesions in ultrafast screening MRI using deep convolutional neural networks

**DOI:** 10.1007/s00330-023-10184-3

**Published:** 2023-09-02

**Authors:** Xueping Jing, Monique D. Dorrius, Sunyi Zheng, Mirjam Wielema, Matthijs Oudkerk, Paul E. Sijens, Peter M. A. van Ooijen

**Affiliations:** 1 Department of Radiation Oncology, and Data Science Center in Health (DASH), Machine Learning Lab, University Medical Center Groningen, University of Groningen, Hanzeplein 1, 9713 GZ Groningen, The Netherlands; 2 Department of Radiology, University Medical Center Groningen, University of Groningen, Hanzeplein 1, 9713 GZ Groningen, The Netherlands; 3 School of Engineering, Artificial Intelligence and Biomedical Image Analysis Lab, Westlake University, No.18 Shilongshan, Road Cloud Town, Xihu District, Hangzhou, 310024 Zhejiang China; 4 Faculty of Medical Sciences, University of Groningen, and Institute of Diagnostic Accuracy, Wiersmastraat 5, 9713 GH Groningen, The Netherlands

**Keywords:** Breast neoplasms, Early detection of cancer, Deep learning, Magnetic resonance imaging

## Abstract

**Objectives:**

To develop a deep learning–based method for contrast-enhanced breast lesion detection in ultrafast screening MRI.

**Materials and methods:**

A total of 837 breast MRI exams of 488 consecutive patients were included. Lesion’s location was independently annotated in the maximum intensity projection (MIP) image of the last time-resolved angiography with stochastic trajectories (TWIST) sequence for each individual breast, resulting in 265 lesions (190 benign, 75 malignant) in 163 breasts (133 women). YOLOv5 models were fine-tuned using training sets containing the same number of MIP images with and without lesions. A long short-term memory (LSTM) network was employed to help reduce false positive predictions. The integrated system was then evaluated on test sets containing enriched uninvolved breasts during cross-validation to mimic the performance in a screening scenario.

**Results:**

In five-fold cross-validation, the YOLOv5x model showed a sensitivity of 0.95, 0.97, 0.98, and 0.99, with 0.125, 0.25, 0.5, and 1 false positive per breast, respectively. The LSTM network reduced 15.5% of the false positive prediction from the YOLO model, and the positive predictive value was increased from 0.22 to 0.25.

**Conclusions:**

A fine-tuned YOLOv5x model can detect breast lesions on ultrafast MRI with high sensitivity in a screening population, and the output of the model could be further refined by an LSTM network to reduce the amount of false positive predictions.

**Clinical relevance statement:**

The proposed integrated system would make the ultrafast MRI screening process more effective by assisting radiologists in prioritizing suspicious examinations and supporting the diagnostic workup.

**Key Points:**

• *Deep convolutional neural networks could be utilized to automatically pinpoint breast lesions in screening MRI with high sensitivity*.

• *False positive predictions significantly increased when the detection models were tested on highly unbalanced test sets with more normal scans*.

• *Dynamic enhancement patterns of breast lesions during contrast inflow learned by the long short-term memory networks helped to reduce false positive predictions*.

**Supplementary Information:**

The online version contains supplementary material available at 10.1007/s00330-023-10184-3.

## Introduction

As the most sensitive breast imaging modality [1], MRI has been used as a supplement tool for high-risk population-based screening where mammography alone is not sufficient [2, 3]. However, the high cost, low availability, and lack of dedicated radiologists restrict the application of MRI for an intermediate- or even low-risk population [4].

Shortening the protocol has been attempted to increase the cost-effectiveness of breast MRI screening [5]. Recently, several innovative abbreviated MRI protocols have been proposed and evaluated [6–9]. Mann et al investigated the feasibility of using ultrafast breast MRI as a standalone technique for breast MRI screening [10]. A multi-reader study showed that this time-resolved angiography with stochastic trajectories (TWIST) sequence-based protocol performed similarly to the full diagnosed protocol and significantly higher screening specificity [11]. Compared with the protocol proposed by Kuhl et al [9], the ultrafast MRI-based protocol produces twenty high spatial acquisitions within 102 s, which allows not only morphologic analysis, but also kinetic analysis during contrast agent inflow. Recent researches also showed the advantages of the early-stage dynamic information in the ultrafast MRI [12, 13].

Automatic detection of breast lesions has the potential to boost the efficiency of screening given that the majority of screening MRIs are lesion free. However, this requires the detection model being highly accurate and dependable. Lesion detection algorithms were developed and tested in prior studies using highly enriched datasets [14–16], where the proportion of scans with suspicious lesions (58.3 to 100%) and cancers (5.7 to 63.8%) is much higher than previously reported. According to a prospective observational study conducted by Kuhl et al [2], a total of 3861 screening MRIs resulted in 61 cancers, 175 BI-RADS 3 and 171 BI-RADS 4 or 5 diagnoses. This yielded a cancer rate of 1.6% and a lesion rate of 9.0%, and both were significantly lower than the rates aforementioned. To ensure the reliability of detection models, it is crucial to test them in a real-world screening setting. The difference in the proportion of scans with and without suspicious lesions between the model development dataset and actual clinical practice can potentially result in misleading performance in lesion detection, thereby diminishing the model’s dependability within the screening population.

In this study, a deep learning–based detection system was developed to identify enhanced lesions in ultrafast MRI. To simulate the screening situation, the detection system was evaluated with test cohorts that had mostly normal examinations. The proposed detection systems aimed to accelerate the screening process by prioritizing MRI scans and reducing the radiologist’s workload.

## Materials and methods

### Patients

The breast MRI scans were retrospectively collected at the University Medical Center Groningen. The institute’s local ethics committee approved this retrospective study (METc 2018/652) and the need for informed consent was waived. The same dataset was used in our earlier work, where a classification system was invented and tested [17]. Lesions in those MRI scans were delicately annotated with bounding boxes to develop a detection system in this study.

To be specific, out of 809 consecutive women who underwent breast MRI examinations at our institute between April 2016 and October 2019, 1447 examinations were first acquired. Details of the acquisition protocols are provided in the Electronic Supplementary Material. Examinations were then included if the following conditions are satisfied: (1) a complete scan contains TWIST sequences; (2) the indication for MRI should be either screening or preoperative assessment; and (3) the identified lesions should have been biopsied or had at least a 2-year follow-up to serve as a gold standard for benignancy. Breast MRIs without TWIST sequences or performed for other reasons (chemotherapy response evaluation, post-surgery follow-up, and implant check) were excluded. Similar with previous research [14], in this study, the left and right breasts of each woman are considered different data points and involved in the training and validation independently.

### Data preprocessing and annotation

**Fig. 1:**
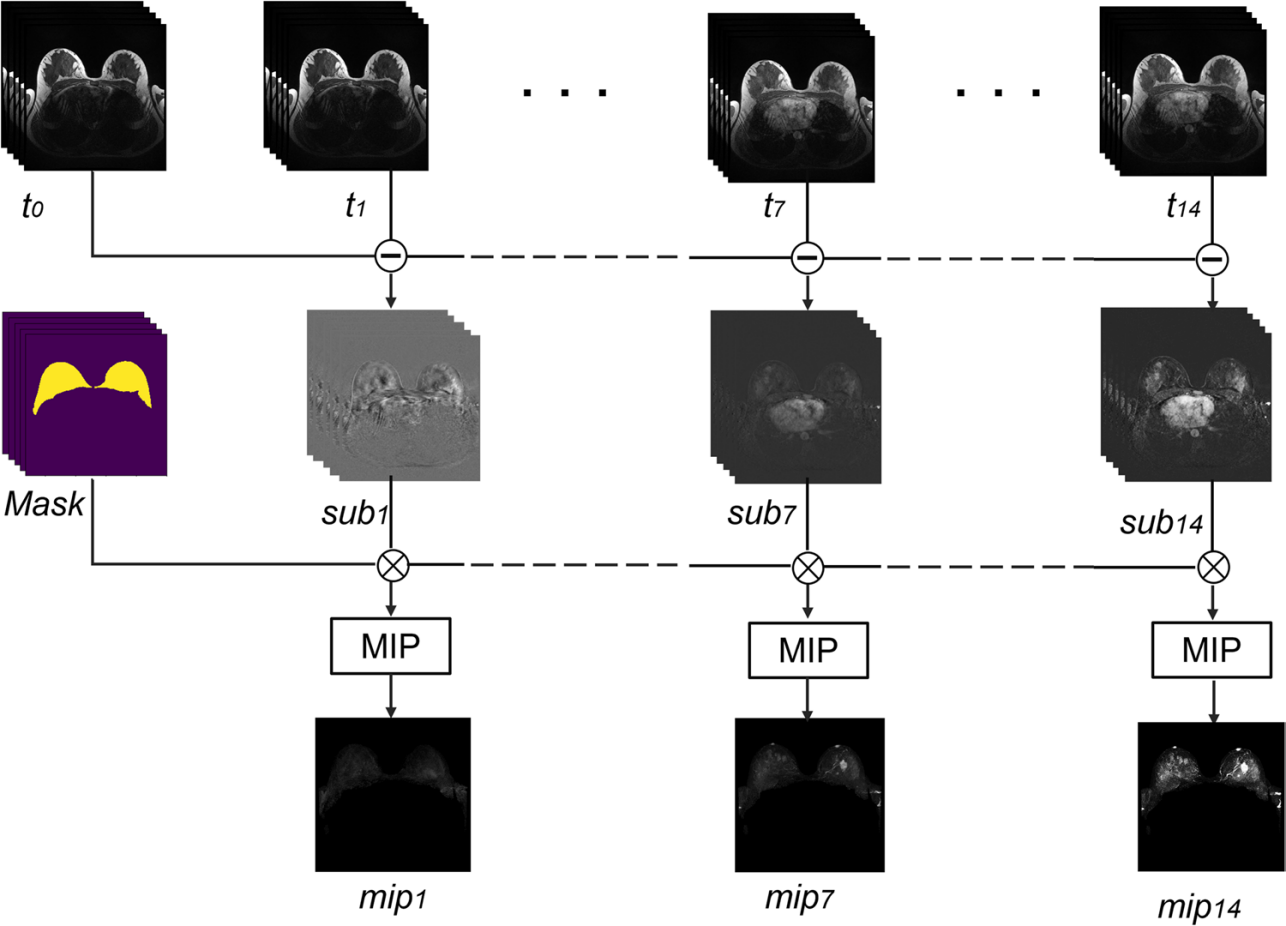
Overview of the image preprocessing pipeline. The pre-contrast acquisition (*t0*) was subtracted by each post-contrast acquisitions (*t1* − *t14*) in the time-resolved angiography with stochastic trajectories (TWIST) sequence to generate corresponding subtraction volumes (*sub1* − *sub14*). The mask from the 3D-Unet was then multiplied with the subtraction volumes to help remove redundant background area. Maximum intensity projection (MIP) operation was then applied to the segmented subtraction volumes to get the MIP images

An overview of the preprocessing procedure of the breast MRI is shown in Fig. 1. A 3D-Unet was used to segment the breast area on the T1-weighted acquisition acquired prior to contrast agent injection to remove the redundant area behind the chest wall and the artifacts surrounding the breasts [18]. Scaling and FOV alignment were used to deploy the 3D masks to TWIST volumes, resulting in segmented volumes. Subtracted volumes were then created by subtracting the segmented pre-contrast volume from the segmented post-contrast volumes. Maximum intensity projection (MIP) images were then generated by applying the MIP operation to the segmented subtracted volumes. The MIP images were then split in two, to produce separate left and right breast images. On the MIP images, lesions were then annotated with bounding boxes (X.J., 3 years of experiences). LabelMe was used for the annotation process [19]. The location of the lesions was derived from the radiology clinical reports and confirmed by an experienced breast radiologist (M.D., 10 years of experience) when in doubt.

### Breast lesion detection with YOLOv5

**Fig. 2:**
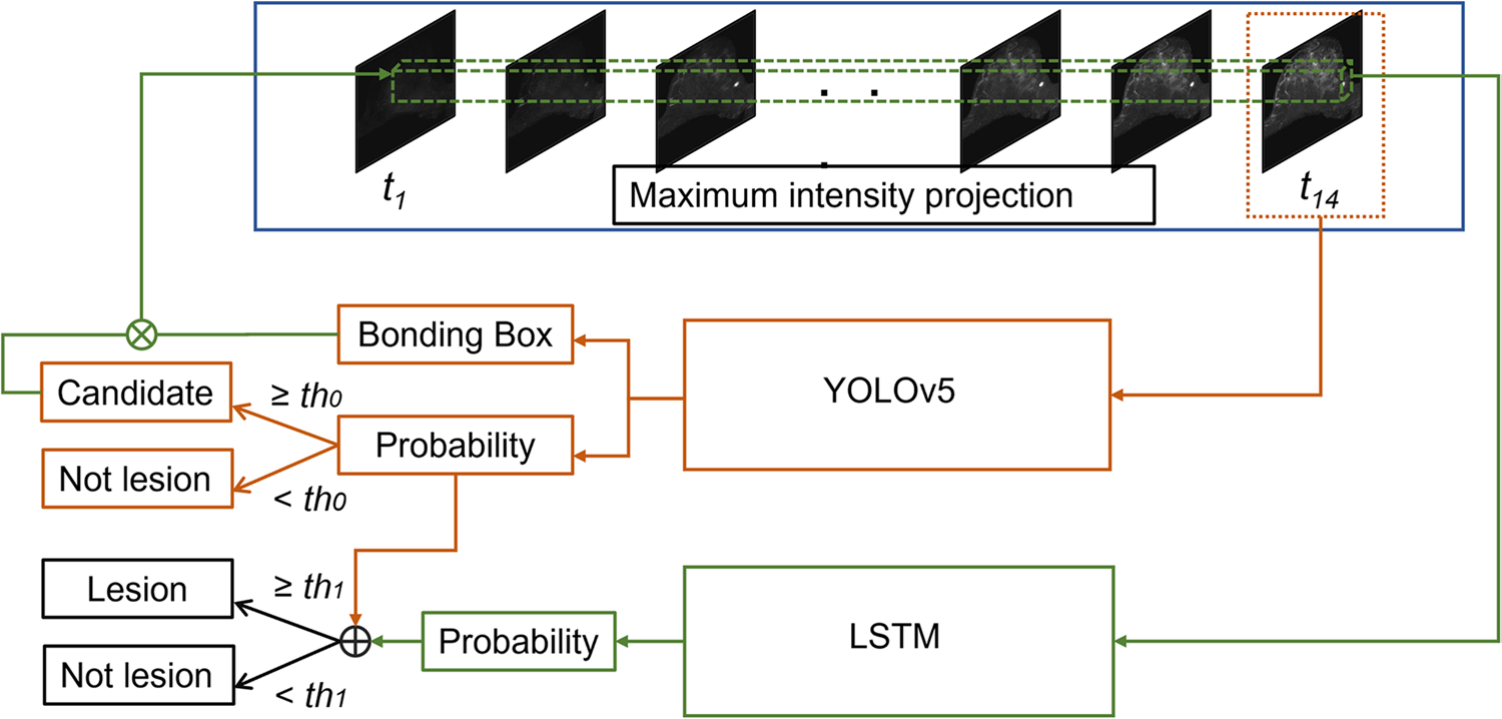
Pipeline of the proposed detection system

The proposed integrated system for detecting breast lesions in ultrafast MRI was composed of two parts: a YOLO model for lesion detection and a long short-term memory (LSTM) network for false positive reduction. The pipeline of the proposed detection method is illustrated in Fig. 2.

YOLO is a one-stage, global context informed architecture that could achieve faster execution speed without compromising the prediction accuracy [20]. Moreover, the YOLO architecture, which allows the use of pure negative images (images devoid of any target object) as input for training, matches the purpose of training models with normal breast images to reduce false positive predictions in a screening setting. MIP images generated in the data preprocessing were used to finetune YOLOv5 model that had been pretrained using the COCO dataset. Three YOLOv5 models (v5n with only 1.9 million parameters, v5m with 21.1 million parameters, and v5x with 86.7 million parameters) were selected and compared in this study to investigate the impact of model size on detection performance. To further investigate the impact of magnetic field strength on models’ detection performance, a subgroup analysis was also conducted in which 1.5-T and 3-T scans were used to train and test YOLOv5 models separately. The detailed information about model finetuning is described in Electonic Supplementary Material.

Benign lesions usually take longer than malignant lesions to be enhanced in the TWIST sequences [12, 21]. To ensure the detectability of benign lesions, MIP images of the early acquisitions in the TWIST were not involved in the development of the detection model. For each ultrafast MRI examination, only the MIP images of the last acquisition were used for the training and validation of the YOLO models.

### False positive reduction

Rather than deducing the results from the YOLO models directly, the locations of the positive predictions with a probability above *th0* from the output of YOLOv5 models were utilized to extract clips across the TWIST sequences. During inference, the LSTM network took each predicted bounding box’s clip (the area spanning the 14 yield MIP images of the ultrafast DCE sequences) as input. The output was a likelihood score of a breast containing a lesion. The predict scores of the YOLOv5 and LSTM networks were then merged to reach the final judgment (Fig. 2). The architecture and training process of the LSTM models are provided in the Electronic Supplementary Material.

### Experiments

Five-fold cross-validation was performed at the breast level to train and evaluate each model and the integrated system. Especially, to avoid data overlap between training and test data from the same breast in patients who had multiple examinations, a group-based shuffle split method was used to ensure data from different dates of the same breast are bound together. Moreover, the YOLO and LSTM networks were trained with the same data splits and then integrated together to prevent data leakage throughout the pipeline.

The number of samples with and without lesions in this analysis was inevitably imbalanced. To overcome this imbalance, all positive samples (breasts with lesions) were merged with an equivalent amount of randomly selected negative samples (breasts without lesions) to establish a balanced development dataset. The rest of the negative samples were subsequently grouped as an isolated negative set and were not used for the model training. However, in addition to assessing the model trained with the balanced dataset, the isolated negative set was also merged to the test data during validation to evaluate the proposed system’s performance with a normal screening prevalence. The performance with and without the isolated negative set was compared. A diagram of the data split and validation set formation is shown in Electronic Supplementary Material Fig. S3.

### Data analysis

Free-response operating characteristic (FROC) analysis was adopted to assess the performance of the evaluated YOLOv5 models and the integrated system. In this study, a false positive prediction is defined as a non-lesion area that is predicted to have a lesion, while a false negative prediction is defined as a lesion that deep learning models failed to detect. To illustrate the effectiveness of the LSTM network for false positive reduction, the sensitivity of the YOLO models alone and the integrated system (YOLO + LSTM) at 0.125, 0.25, 0.5, 1, and 2 false positives per breast were also calculated. Meanwhile, the detection ability of each model to detect malignant lesions was analyzed in the same way. The data analysis was performed with Scikit-learn 0.22.1 and COCO-FROC-analysis 0.2.0 packages in Python programming language.

## Results

### Included lesions

A total of 488 women were included in this study. The mean age of the included women (*n* = 488) was 48.5 years (range, 27–83 years), for women with breast lesions (*n* = 133) was 52.0 years (range, 27–83 years), and for women with malignant lesions (*n* = 58) was 57.6 years (range, 34–83 years). Five women were undergoing MRI examination with an indication of preoperative assessment, and all had malignant lesions, which account for 8.6% of the total number of women with malignant lesion and 3.8% of women with lesion. A flowchart of this process is illustrated in Fig. S4; further details of these patients have previously been reported [17].

In total, 962 single breasts were derived from the included patients; 14 breasts were excluded due to mastectomy. For included breasts, 83.0% (*n* = 799) were reported as lesion free, and 315 were derived from 1.5-T scans and 484 were derived from 3.0-T scans. Seventeen percent (*n* = 163) had at least one lesion, in which 7.3% (*n* = 70) contained only one lesion, and 9.7% (*n* = 93) contained multiple lesions. In total, 265 lesions were annotated, 71 lesions were derived from 56 1.5-T scans and 194 were derived from 112 3.0-T scans. The median size of all reported lesions was 13.0 mm (range, 5.0–110.0 mm), 9.0 mm (range, 5.0–81.0 mm) for benign lesions (*n* = 190) and 22.0 mm (range, 6.0–110.0 mm) for malignant lesions (*n* = 75). The detailed information of the lesions is illustrated in Table 1.

**Table 1:** Characteristics of the included lesions

Characteristics	Value	Size (mm)
Benign lesions	190	13.4 (5–81)
Adenosis	74	8.5 (5–15)
Fibroadenoma	21	18.8 (9–48)
Hyperplasia	7	21.2 (6–81)
Cyst	3	9.0 (6–12)
Inflammation	2	46.0 (32–60)
Other*	83	15.1 (6–51)
Malignant lesions	75	27.6 (6–110)
Invasive ductal carcinoma	57	25.6 (6–76)
Invasive lobular carcinoma	5	49.8 (13–110)
Ductal carcinoma in situ	7	29.5 (7–62)
Micropapillary carcinoma	2	14.5 (13–16)
Apocrine carcinoma	1	24
Mucinous carcinoma	2	45 (8–82)

^*^Lesions were indicated in the radiology reports but not biopsied or differentiated by radiologists

### YOLOv5 model performance

During cross-validation, the fine-tuned YOLOv5x model showed a sensitivity of 0.95 (0.85–1.0), 0.97 (0.91–1.0), 0.98 (0.93–1.0), and 0.99 (0.96–1.0) with 0.125, 0.25, 0.5, and 1 false positive per breast, respectively, compared to 0.94 (0.91–0.98), 0.97 (0.95–1.0), 0.98 (0.96–1.0), and 0.99 (0.96–1.0) for the YOLOv5m model and 0.77 (0.69–0.86), 0.87 (0.78–0.94), 0.93 (0.89–0.98), and 0.98 (0.94–1.0) for the YOLOv5n model. With two false positives per breast, all models obtained a sensitivity of 1.0 for lesion identification. For different magnetic field strength subgroup, the YOLOv5x model achieved an overall higher sensitivity on the 3.0 T subgroup than the 1.5 T subgroup, with a sensitivity of 0.76 (0.65–0.84) and 0.69 (0.59–0.83) with 0.125 false positives per breast, respectively. Detailed results of the model on each subgroup are provided in the Electronic Supplementary Material.

**Fig. 3:**
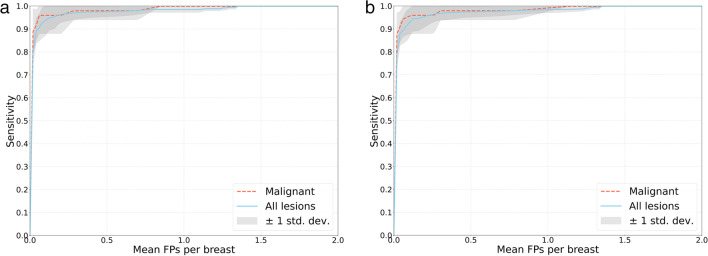
Free-response operating characteristic curves of the (**a**) YOLOv5x model and (**b**) the integrated system for all lesions and malignant lesions during cross-validation

For malignant lesion detection, the YOLOv5x model had a sensitivity of 0.96 (0.80–1.0), 0.97 (0.86–1.0), 0.98 (0.90–1.0), and 1.0 with 0.125, 0.25, 0.5, and 1 false positive per breast, respectively, compared to 0.97 (0.90–1.0), 1.0, 1.0, and 1.0 for the YOLOv5m model and 0.94 (0.90–1.0), 0.97 (0.92–1.0), 0.98 (0.92–1.0), and 0.98 (0.92–1.0) for the YOLOv5n model. All models had a sensitivity of 1.0 for malignant lesion detection, with two false positives per breast. The FROC curve of the YOLOv5x models is shown in Fig. 3a.

### False positive reduction with LSTM

To reduce false positive predictions, an LSTM network was used to further identify candidates from the output of the YOLOv5 models. The performance of the integrated system was evaluated using the same data split as the single YOLO model during cross-validation. The FROC curve of the integrated system is shown in Fig. 3b. The integrated system retained approximately equivalent sensitivity for both malignant and all lesions detection, after integrating the LSTM network (Table 2). To demonstrate the efficacy of the false positive reduction, Table 3 shows the number of false positive predictions of the YOLOv5x model in each fold of the cross-validation with and without the LSTM network. The findings were obtained using a 0.5 *th0* and 0.5 *th1* threshold setting.

**Table 2:** Sensitivity of each model for all lesions and malignant lesions with different average numbers of false positives per breast

Model	False positive per breast									
	0.125		0.25		0.5		1		2	
	All lesion	Malignant	All lesion	Malignant	All lesion	Malignant	All lesion	Malignant	All lesion	Malignant
YOLOv5n	0.77 (0.69–0.86)	0.94 (0.90–1.0)	0.87 0.78–0.94	0.97 (0.92–1.0)	0.93 (0.89–0.98)	0.98 (0.92–1.0)	0.98 (0.94–1.0)	0.98 (0.92–1.0)	1.0 (-)	1.0 (-)
YOLOv5m	0.94 (0.91–0.98)	0.97 (0.90–1.0)	0.97 (0.95–1.0)	1.0 (-)	0.98 (0.96–1.0)	1.0 (-)	0.99 (0.96–1.0)	1.0 (-)	1.0 (-)	1.0 (-)
YOLOv5x	0.95 (0.85–1.0)	0.96 (0.80–1.0)	0.97 (0.91–1.0)	0.97 (0.86–1.0)	0.98 (0.94–1.0)	0.98 (0.90–1.0)	0.99 (0.97–1.0)	1.0 (-)	1.0 (-)	1.0 (-)
YOLOv5n + LSTM	0.76 (0.69–0.84)	0.94 (0.90–1.0)	0.86 (0.77–0.93)	0.97 (0.92–1.0)	0.93 (0.89–0.97)	0.98 (0.92–1.0)	0.98 (0.93–1.0)	0.98 (0.92–1.0)	1.0 (-)	1.0 (-)
YOLOv5m + LSTM	0.94 (0.90–0.97)	0.96 (0.90–1.0)	0.97 (0.94–1.0)	1.0 (-)	0.98 (0.96–1.0)	1.0 (-)	0.99 (0.96–1.0)	1.0 (-)	1.0 (-)	1.0 (-)
YOLOv5x + LSTM	0.95 (0.85–1.0)	0.96 (0.80–1.0)	0.96 (0.90–1.0)	0.96 (0.80–1.0)	0.98 (0.94–1.0)	0.98 (0.90–1.0)	0.98 (0.96–1.0)	0.99 (0.96–1.0)	1.0 (-)	1.0 (-)

Number in parentheses are the range of sensitivity. *LSTM*, long short-term memory network

**Table 3:** Performance of the YOLOv5x model and the integrated system in each fold

Fold	Number of breasts*	Without LSTM network		With LSTM network		False positive reduction
		Sensitivity	PPV	Sensitivity	PPV	
Fold 0	702 [33 + 669]	0.98 (52/53)	0.20 (52/248)	0.98 (52/53)	0.23 (52/228)	0.10 (20)
Fold 1	700 [32 + 668]	0.95 (42/44)	0.17 (42/252)	0.95 (42/44)	0.18 (42/229)	0.11 (23)
Fold 2	700 [32 + 668]	1 (59/59)	0.26 (59/227)	1 (59/59)	0.29 (59/204)	0.14 (23)
Fold 3	700 [32 + 668]	1 (49/49)	0.24 (49/205)	1 (49/49)	0.28 (49/178)	0.17 (27)
Fold 4	700 [32 + 668]	0.90 (54/60)	0.24 (54/222)	0.87 (52/60)	0.28 (52/184)	0.23 (38)
Average	–	0.97 (0.90–1.0)	0.22 (0.17–0.26)	0.96 (0.87–1.0)	0.25 (0.18–0.29)	0.15 (0.10–0.23)

Number in parentheses are the number of lesions. *Number in brackets is the number of breasts with lesion plus number of breasts without lesion

*PPV*, positive predictive value; *LSTM*, long short-term memory network

**Fig. 4:**
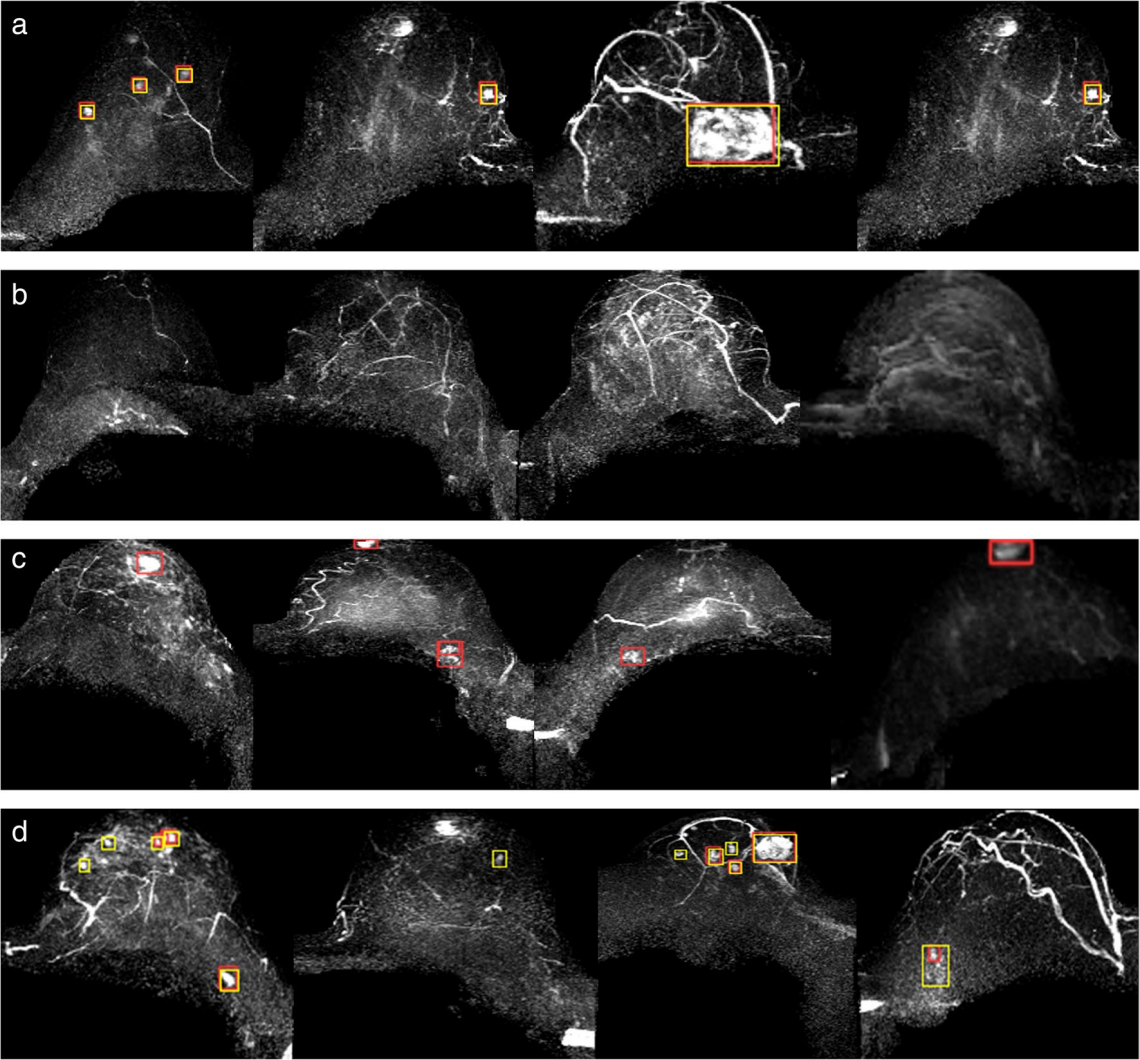
Example of (**a**) true positive, (**b**) true negative, (**c**) false positive, and (**d**) false negative detection result of the integrated system. The wrongly predicted tissues in **c** (from left to right): nipple, nipple and lymph node, lymph node, nipple. The false negative predictions in **d** (from left to right): foci, adenosis, biopsy-confirmed invasive ductal carcinoma, and biopsy-confirmed adenocarcinoma with incorrect location estimation (Yellow boxes indicate the ground truth; red boxes present the predictions from the integrated system.)

Example of final detection results of the integrated system is shown in Fig. 4. Only detections with probability above the threshold and with correct location estimates deemed as true positives (Fig. 4a), while normal breast tissues without any lesion predictions deemed as true negatives (Fig. 4b). Other tissues, such as nipples, lymph nodes, vessels, and enhanced parenchyma, which were incorrectly predicted as lesions were considered false positives. Any missed lesions and lesions with incorrect location estimations were considered as false negatives.

## Discussion

In this study, we developed a deep learning–based method for automated detection of breast lesions for the purpose of breast cancer screening. Unlike previous research on breast MRI lesion detection, we focused on the performance of models with a screening prevalence. The model was evaluated using test sets that included mostly normal scans rather than only scans with lesions. The proposed integrated system had a sensitivity that was comparable to previous studies while producing fewer false positives.

To address the high false positive rate for breast lesion detection, an LSTM network aimed at identifying different dynamic intensity patterns during contrast inflow was employed. Using the YOLOv5x model as an example, the LSTM network could help eliminate 15% of the false positive predictions. This LSTM network operates on the output of the YOLOv5 models and has the potential to misclassify correctly predicted lesions, resulting in a decreased sensitivity. However, despite a few misclassifications induced by the LSTM network, the integrated system retained its high sensitivity in general during cross-validation, and the feared significant decline in sensitivity was not observed (Fig. 3b and Table 3).

This is a follow-up study of our previous work in which a classification system was invented to identify lesion-free scans that use only TWIST sequences [17]. However, the previous study mainly focused on the primary tumors in each breast and take no account of minor findings. Instead of generating a categorical prediction, we elaborately annotated all lesions with bounding box in the cohort and trained detection methods to localize lesions in the breasts, for the propose of providing more precise and visible results. Compared with the classification system in our previous work, which focused on excluding normal scans to minimize the reading list, the detection model developed in this study gives more immediate visual assistance, allowing radiologists to focus on the suspicious breast lesions directly.

The size of YOLOv5 models has impact on the detection performance. Three different YOLOv5 models were evaluated in this study. Compared with the 0.77 sensitivity achieved by the YOLOv5n model, both YOLOv5m and YOLOv5x achieved superior sensitivity (0.94 and 0.95) with 0.125 false positives per breast. This advantage gradually vanished with a higher false positive rate. We also investigate the impact of magnetic field strength on detection models’ performance. The YOLOv5x model achieved an overall higher sensitivity on the 3.0 T subgroup than the 1.5 T subgroup. However, despite the model’s poor performance, which likely resulted by the insufficient amount of positive training samples in each subgroup, it is difficult to draw the conclusion that a stronger magnetic field benefits the model’s performance. Collecting more data would enable us to conduct a comprehensive subgroup analysis.

The integrated system is developed to detect all enhanced breast masses (> 5 mm), not just malignant ones. Several previous studies developed and tested models for cancer detection in DCE-MRI and were successful [22, 23]. However, the diagnosis of malignancy is heavily dependent on additional MRI sequences and even biopsies. Using a single model to locate and identify just malignancies while ignoring other suspicious findings is risky and unreasonable, especially for screening purposes, dedicated AI models may be more useful for malignancy identification of candidate lesions [24].

One should keep in mind is that ultrafast MRI has not yet been evaluated in a real screening cohort [25]. Studies that compared the effectiveness of ultrafast and conventional MRI were also mainly retrospective studies involving patients with lesions in MRI. Meanwhile, the ultrafast MRI techniques are not standardized yet; parallel imaging, viewing sharing, and compressed sensing are all used to obtain a higher temporal resolution but are all referred to ultrafast MRI [26, 27].

This study has several limitations. One of the limitations was that there was no independent test set, instead, we used cross-validation to illustrate the effectiveness of the integrated system. Meanwhile, due to the rarity of the number of cancers in a screening population and the large amount of data required to train deep learning models, preoperative MRI scans were also included in the dataset to increase the number of cancers. Hence, the system should be further evaluated with additional dedicated multicenter multivendor databases. Another limitation is that only part of benign lesions was histologically examined; this reflected the radiologists’ confidence in their ability to determine the need for biopsy based on imaging outcomes.

This study demonstrates the ability of a deep learning–based method to detect candidate findings in ultrafast breast MRI. This proposed fully automated method could be helpful in detecting breast lesions in the setting of breast cancer screening, thereby potentially reducing radiologists’ workload. This, in turn, will allow breast MRI screening to apply to a larger population, resulting in better preventive health care delivery.

### Supplementary Information

Below is the link to the electronic supplementary material.

Supplementary file1 (PDF 279 kb)

## Abbreviations

FROC: Free-response operating characteristic
LSTM: Long short-term memory
MIP: Maximum intensity projections
PPV: Positive predictive value
TWIST: Time-resolved angiography with stochastic trajectories

## Declarations

### Guarantor

The scientific guarantor of this publication is Peter M. A. van Ooijen.

### Statistics and biometry

No complex statistical methods were necessary for this paper.

### Study subjects or cohorts overlap

Some study subjects or cohorts have been previously reported in Jing X, Wielema M, Cornelissen LJ, et al (2022) Using deep learning to safely exclude lesions with only ultrafast breast MRI to shorten acquisition and reading time. *Eur Radiol*. https://doi.org/10.1007/s00330-022-08863-8.

### Methodology

• Retrospective

• Diagnostic or prognostic study

• performed at one institution
