# Supplementary material for: Localization of contrast-enhanced breast lesions in ultrafast screening MRI using deep convolutional neural networks
Source: Eur Radiol. 2023 Sep 2;34(3):2084–92. doi: 10.1007/s00330-023-10184-3 (PMC10873226; doi:10.1007/s00330-023-10184-3)
Supplement: Supplementary file 1 — Supplementary file1 (PDF 279 kb) [file 330_2023_10184_MOESM1_ESM.pdf]

Supplementary Materials

MRI protocol

All DCE-MRI examinations acquired were performed with a full diagnostic protocol on either a 3.0 T or 1.5 T scanner (MAGNETOM Skyra or MAGNETOM Avantofit, Siemens Healthineers) in prone position. The full diagnostic protocol included T1-weighted high spatial resolution DCE-MRI, high temporal resolution DCE imaging (TWIST sequence), T2-weighted imaging, and diffusion-weighted imaging (DWI). For the 1.5 T scanner, the parameters of the TWIST sequence were as follow: repetition time (TR): 2.5 ms; echo time (TE): 0.9 ms; flip angle: 20 degrees; phase oversampling: 26%; slice oversampling: 20%; voxel size: 0.68 mm × 0.68 mm × 3.0 mm; temporal resolution: 5.2 seconds, field of view (FOV): 350 mm and without fat suppression. For the 3.0 T scanner, the parameters were set as: TR: 4.12 ms; TE: 2.06 ms; flip angle: 20 degrees; phase oversampling: 20%; slice oversampling: 0%; voxel size: 0.91 mm × 0.91 mm × 3.0 mm; temporal resolution: 4.3 seconds, FOV: 350 mm and without fat suppression. The full protocol takes 17.95 and 19.61 minutes on 3.0 T and 1.5 T scanners, respectively, while the 15 acquisitions in the TWIST sequence take 1.3 and 1.46 minutes, respectively.

The training process of the YOLOv5 models an LSTM model.

For the training of the YOLOv5 models, the classes of the object were set to 1. The Adam optimizer was used, with a learning rate of 10^{-4}, and the loss function was the combination of objectness score, class probability score, and bounding box intersection over union loss. The number of finetuning epochs was 60. We followed the instruction of the original implementation and used pretrained models provide by Ultralytics (https://github.com/ultralytics/yolov5).

For the training of the LSTM network (Fig S1), the cropped regions of the annotated lesions were utilized as positive examples. Negative examples were obtained by randomly cropping the same position area with the same size from the breasts without lesions (Fig S2). Patients with lesions were not involved in creating negative examples. To feed into the model, the cropped area was resized to 128×128×14 during training. A pre-trained ResNet-18 model was used as a feature extractor of the LSTM network. An Adam optimizer with an initial learning rate of 10^{-4} was used for model training.

The binary cross-entropy was used as the loss function. The number of training epochs was 60. Random horizontal flip (probability of 0.5) and random rotation (within 10°) were applied to all clips randomly for data augmentation.

Comparison of 3.0 T and 1.5 T subgroup

To compare the detection performance on 3T and 1.5T subgroups, the YOLOv5x model were trained and tested with each subgroup separetly. Following the same cross validation stratiegy, the YOLOv5x models had a sensitivity of 0.76 (0.65-0.84), 0.85 (0.75-0.95), 0.92 (0.88-1.0) and 1.0 with 0.125, 0.25, 0.5 and 1 false positive per breast, respectively on the 3.0 T subgroup, and a sensitivity of 0.69 (0.59-0.83), 0.71 (0.60-0.84), 0.72 (0.60-0.84) and 0.73 (0.60-0.86) with 0.125, 0.25, 0.5 and 1 false positive per breast, respectively on the 1.5 T subgroup. For malignant lesion detection, a sensitivity of 0.92 (0.83-1.0), 0.97 (0.83-1.0), 0.97 (0.83-1.0), 0.97 (0.83-1.0) and 0.64 (0.5-0.81), 0.66 (0.53-0.81), 0.69 (0.56-0.87), 0.72 (0.56-0.89) was achieved with 0.125, 0.25, 0.5 and 1 false positive per breast on 3.0 T and 1.5 T subgroups, respectavely. A sensitivity of 1.0 for both lesion and malignancy detection was achieved, with 2 false positive per breast on each subgroup.

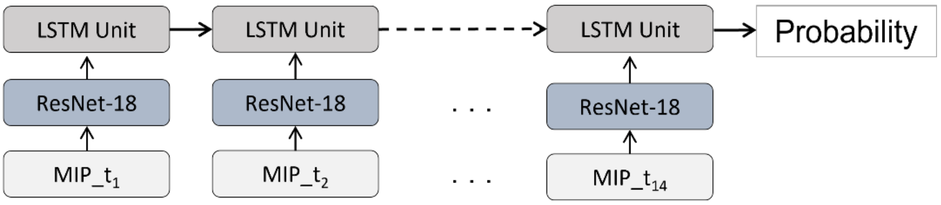

Figure S1. Diagram of the LSTM network. A ResNet-18 model takes Maximum intensity projection (MIP) images from each time point ($t_1, t_2, \ldots, t_{14}$) as input and forwards extract features to the Long short-term memory (LSTM) Unit. The last LSTM Unit produce a probability of the detection (from YOLO) as true positive.

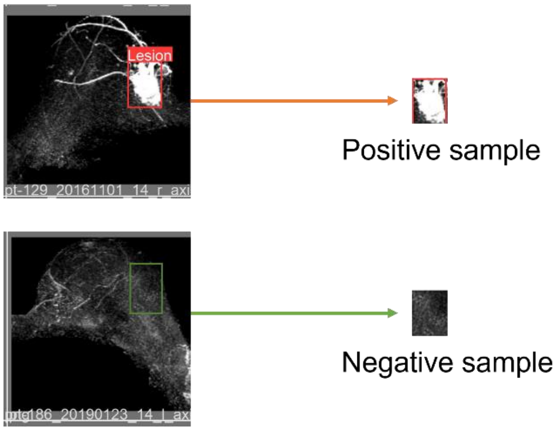

Figure S2. The examples of positive and negative samples generated for the training of the LSTM model. For each positive sample, a pure negative image was randomly selected and a negative sample was extract with the same size at the same location.

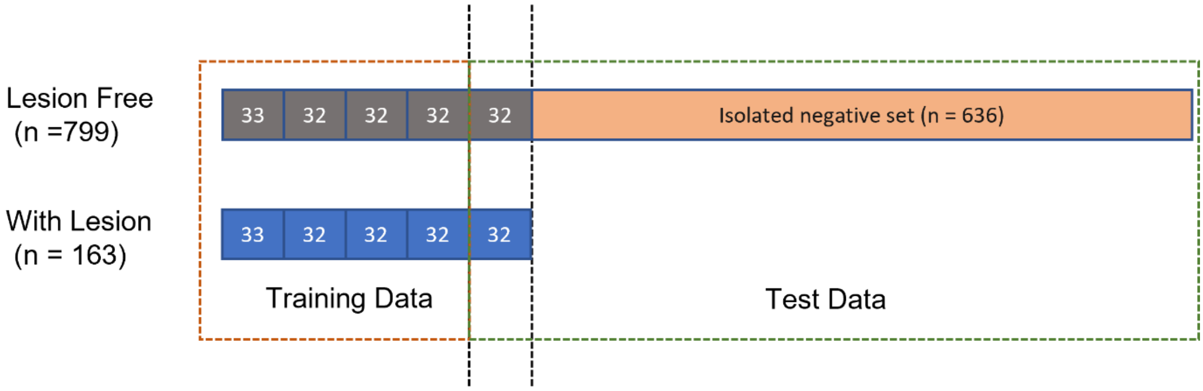

Figure S3. The data split and validation set formation diagram. All breasts with lesions and the same amount of breasts without lesions were split into 5 fold. For the training, 4 folds with equal number of breasts with lesion and lesion free breasts were used for the training of YOLO and LSTM models, while testing, the rest fold combined with the isolated negative set were used to validate the performance of the system.

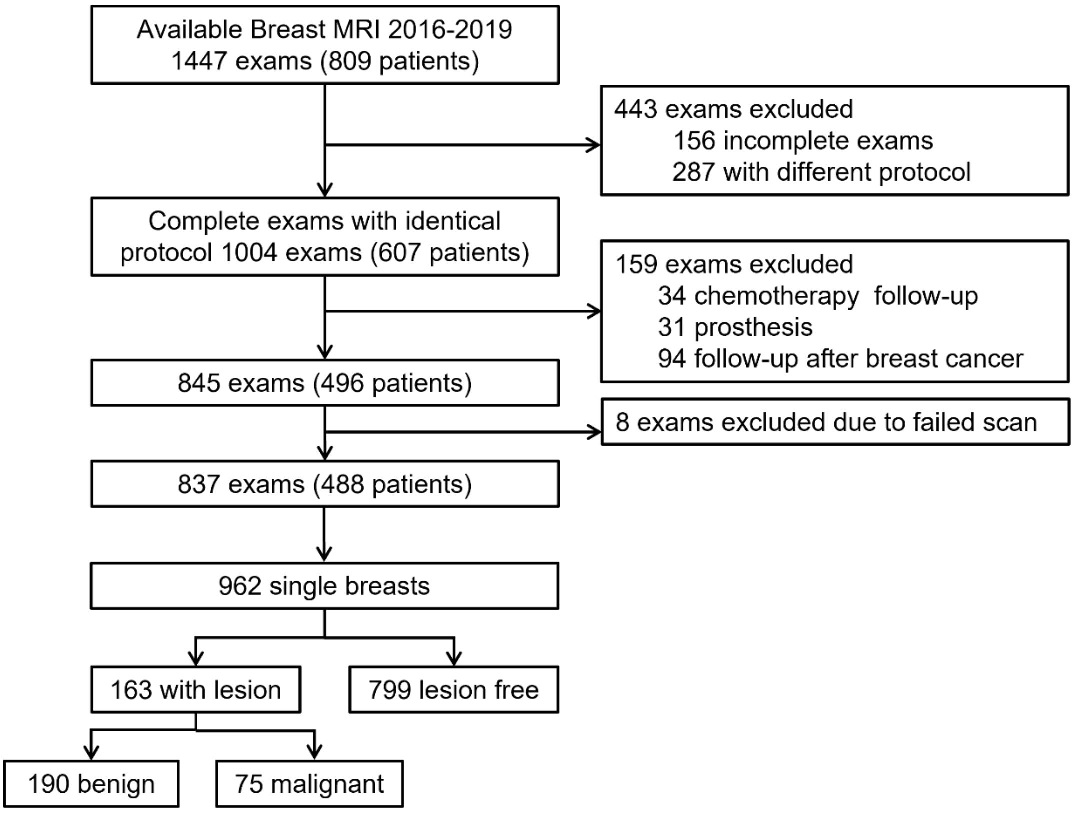

Figure S4. The flowchart of patient selection.
